# Qiguiyin Decoction Improves Multidrug-Resistant *Pseudomonas aeruginosa* Infection in Rats by Regulating Inflammatory Cytokines and the TLR4/MyD88/NF-*κ*B Signaling Pathway

**DOI:** 10.1155/2022/5066434

**Published:** 2022-01-13

**Authors:** Guochao Chen, Wanqiao Zhang, Lingbo Kong, Chengxiang Wang, Xiaojing Lai, Xue Yu, Yuying Guo, Jun Wu, Qun Ma

**Affiliations:** ^1^School of Chinese Materia Medica, Beijing University of Chinese Medicine, Beijing 102488, China; ^2^Department of Emergency Medicine, Dongzhimen Hospital Affiliated to Beijing University of Chinese Medicine, Beijing 100700, China; ^3^School of Traditional Chinese Medicine, Beijing University of Chinese Medicine, Beijing 102488, China; ^4^School of Life Sciences, Beijing University of Chinese Medicine, Beijing 102488, China

## Abstract

*Pseudomonas aeruginosa* (PA), a Gram-negative bacterium, has a high detection rate in hospital-acquired infections. Recently, the frequent appearance of multidrug-resistant (MDR) PA strain with high morbidity and mortality rates has aggravated the difficulty in treating infectious diseases. Due to its multiple resistance mechanisms, the commonly used antibiotics have gradually become less effective. Qiguiyin decoction (QGYD) is a clinically experienced prescription of Chinese herbal medicine, and its combined application with antibiotics has been confirmed to be effective in the clinical treatment of MDR PA infection, which could be a promising strategy for the treatment of drug-resistant bacterial infections. However, the mechanism of QGYD restoring antibiotics susceptibility to MDR PA remains unclear. In the present study, we investigated the effects of QGYD and levofloxacin (LEV) singly or in combination on MDR PA-induced pneumonia rat models. Further analysis was carried out in the serum differential expression profiles of inflammatory cytokines by cytokine antibody array. Besides, the lung TLR4/MyD88/NF-*κ*B signaling pathway was detected by RT-qPCR. Our results showed that based on the treatment of MDR PA-infected rat model with LEV, the combination of QGYD improved the general state and immune organ index. Furthermore, it moderately increased the expressions of proinflammatory cytokines including IL-1*β*, IL-6, and TNF-*α* in the early stage of infection and decreased their release rapidly in the later stage, while regulated the same phase change of anti-inflammatory cytokine IL-10. In addition, the adhesion molecule ICAM-1 was significantly downregulated after QGYD combined with LEV treatment. Moreover, the mRNA expressions of TLR4, MyD88, NF-*κ*B, and ICAM-1 were significantly downregulated. These results indicated that the mechanism of QGYD restoring LEV susceptibility to MDR PA was related to its regulation of inflammatory cytokines and the TLR4/MyD88/NF-*κ*B signaling pathway, which provides theoretical support for the clinical application of QGYD combined with LEV therapy to MDR PA infection.

## 1. Introduction

*Pseudomonas aeruginosa* (PA) is one of the most common Gram-negative bacteria that cause hospital-acquired infections, with strong pathogenicity and increasing clinical isolation rate [1]. Levofloxacin (LEV) used to be an effective antibiotic against PA infection [2]. However, with its extensive clinical application, PA has been increasingly resistant to LEV [3–6]. Neither increasing doses, the combination of multiple antibiotics, nor continuous replacement of antibiotics can effectively control drug-resistant PA infection. Recently, multidrug-resistant (MDR) PA strain has appeared frequently with high infection and mortality rates, which poses a great challenge to clinical anti-infection treatment. Therefore, it is particularly urgent to find a more effective therapeutic strategy.

It is recognized that inflammatory immune response plays an important role in the occurrence and development of PA infection [7, 8]. Furthermore, the regulation of inflammatory cytokines has been proved to play a central role in initiating a proper inflammatory response against bacterial infections [9]. Thus, the dynamic change of different inflammatory cytokines may be responsible for the development of PA infection. Moreover, increasing evidence has shown that the TLR4/MyD88/NF-*κ*B signaling pathway is associated with inflammatory immune response by regulating the expression of various inflammatory cytokines such as interleukin-6 (IL-6), interleukin-1 beta (IL-1*β*), and tumor necrosis factor alpha (TNF-*α*) [10, 11]. In addition, it is reported that the TLR4/MyD88/NF-*κ*B signaling pathway is involved in the inflammatory immune response of various diseases [12, 13], and it has been confirmed that many chemicals can exert therapeutic effects by regulating this signaling pathway [14–16]. Therefore, regulating the TLR4/MyD88/NF-*κ*B signaling pathway could be a promising strategy for the prevention and treatment against PA infection.

In the treatment of anti-infection, traditional Chinese medicine has its unique advantages. Studies [17–20] have shown that traditional Chinese medicine can not only inhibit and kill bacteria but also regulate the body immunity and delay or reverse bacterial resistance in treating infectious diseases. Qiguiyin decoction (QGYD) is composed of *Astragalus mongholicus* Bunge, *Angelica sinensis* (Oliv.) Diels, *Lonicera japonica* Thunb., *Reynoutria japonica* Houtt., and *Artemisia annua L*. It is an effective Chinese herbal compound summarized based on years of clinical experience in antidrug-resistant bacteria infection [21]. More importantly, it has been confirmed that QGYD can synergize with antibiotics to inhibit bacteria and improve clinical efficacy [22]. Besides, the previous studies [23–28] have shown that QGYD could delay and reverse the resistance of PA to antibiotics, regulate the inflammation and immune disorder caused by PA infection, and enhance the clinical efficacy of anti-PA infection. However, the mechanism of QGYD restoring LEV susceptibility to MDR PA infection remains unclear.

In the current study, we investigated the efficacy of QGYD and LEV combination therapy on MDR PA-induced pneumonia in rats. Additionally, the mechanism of QGYD restoring LEV susceptibility was explored through the regulation of inflammatory cytokines and the TLR4/MyD88/NF-*κ*B signaling pathway.

## 2. Materials and Methods

### 2.1. Reagents and Chemicals

The main reagents and chemicals used in this experiment are as follows: DNA Marker 3000 plus and 6× DNA Loading Buffer were from Beijing Biodee Biotechnology Ltd. (Beijing, China). GoldView Type I nucleic acid stain and 5× TBE were from Beijing Biotopped Co., Ltd. (Beijing, China). RNase Free Water was from Beijing Solarbio Science Technology Co., Ltd. (Beijing, China). Cytokine Antibody Chip Kit was from RayBiotech, Inc. (Atlanta, USA). Tissue Total RNA Mini Kit was from Magen Co., Ltd. (Guangzhou, China). PrimeScript™ RT Master Mix and TB Green Premix Ex Taq™ II were from Takara Biomedical Technology Co., Ltd. (Beijing, China). Levofloxacin tablets (Batch No: BS015A1) were purchased from Daiichi Sankyo Pharmaceutical Co., Ltd. (Beijing, China).

### 2.2. Animals and Bacterial Suspension

Forty male SD rats (180-220 g) were purchased from SPF Biotechnology Co., Ltd. (Permit No: SCXK (Jing) 2016-0002, Beijing, China) and housed under a normal 12 h dark/light cycles with the temperature controlled at 20-26°C and the relative humidity at 40-70%. All the rats were adaptively fed with a standard diet and water for 3 days before the experiments. This study was carried out following the recommendations of the Guide for Care and Use of Laboratory Animals and Animal Experimental Committee of Beijing University of Chinese Medicine. The protocol was approved by the Animal Experimental Committee of Beijing University of Chinese Medicine.

MDR PA clinical strain (No.1805020) was provided by the Department of Clinical Laboratory of Dongzhimen Hospital, affiliated to Beijing University of Chinese Medicine. The MDR PA strain was inoculated on the solid medium of nutrient agar and plate cultured at 37°C for 24 h. The next day, the MDR PA suspension was adjusted to a concentration of 3 × 10^8^ CFU/mL by a turbidimetric method with sterile saline.

### 2.3. Preparation of QGYD

QGYD extract was provided by School of Chinese Materia Medica, Beijing University of Chinese Medicine. QGYD was composed of five traditional Chinese medicines which were purchased from Beijing Heyanling Pharmaceutical Development Co., Ltd. (Beijing, China), including *Astragalus mongholicus Bunge* (Huangqi), *Angelica sinensis* (Oliv.) Diels (Danggui), *Lonicera japonica* Thunb. (Jinyinhua), *Reynoutria japonica* Houtt. (Huzhang), and *Artemisia annua* L. (Qinghao), with the mixed proportion of the respective compound being 12 : 2 : 3 : 2 : 3. The preparation process of QGYD extract was extracted three times with 12, 10, and 10 folds (*v*/*w*) distilled water, 1 h for each time. The extracted solutions were combined and concentrated to 1.0 g/mL (calculated according to the content of crude drugs) with the concentration of alcohol precipitation 60%. Then, the supernatant was collected and dried in a vacuum at 60°C, and QGYD extract was obtained (yield: 26.35%). The HPLC fingerprint results from three different samples of QGYD extract (S1, S2, and S3) were listed in Supplementary Figure 1 of the Supplementary Materials. In this study, the QGYD extract was dissolved in distilled water and prepared for intragastrical administration.

### 2.4. Animal Model and Experimental Treatments

The rats were randomly divided into five groups (eight rats per group), including the Control group, Model group, LEV group, QGYD group, and Qiguiyin decoction combined with levofloxacin (QGYD-LEV) group. Then, the MDR PA-induced pneumonia rat model was established by tracheal intubation using a venous trocar as endotracheal tube after anesthetized. A volume of 0.2 mL MDR PA suspension was slowly injected into the rat lung through the tracheal intubation, while an equal volume of sterile saline was injected in the same way to the Control group. After modeling, the Control group was fed separately from the MDR PA-infected groups. In this study, the rat dosage was 6.25 times that for a 60 kg adult (equivalent dose). An hour after modeling, the rats were administered with QGYD extract (3.0 g/kg/d) in QGYD group and with LEV (0.03125 g/kg/d) in LEV group twice a day for 5 days by gavage. The QGYD-LEV group rats were given with QGYD extract and LEV at the aforementioned doses, whereas the Control group and model group were given the same amount of distilled water.

The general states including respiratory rate and mental state of the rats were observed daily after modeling, and the body weight was recorded in 5 days. The blood of rats in each group was collected from the eyelids at 3 h, 8 h, 24 h, and 72 h after model establishment, and from the abdominal aorta at 1 h after the last gavage on the 5th day, in addition, the lung, spleen, and thymus samples were collected at the same time. All the collected blood was subsequently placed at 4°C for 3-4 h and centrifuged at 3500 rpm for 10 min; then, the serum was collected and stored at –80°C for further analysis. The left lung was separated and quickly transferred to -80°C until RT-qPCR detection, while the upper lobe of the right lung was separated for the calculation of lung wet weight to dry weight ratio (*W*/*D*). Additionally, the spleen (or thymus) index was calculated by dividing the spleen (or thymus) weight (mg) by the body weight (g).

### 2.5. Determination of Serum Inflammatory Cytokines

The Cytokine Antibody Chip Kit was used to simultaneously detect 10 common inflammatory cytokines including IL-1*β*, IL-6, TNF-*α*, IFN-*γ*, IL-10, ICAM-1, TIMP-1, MCP-1, Leptin, and L-selectin in rat serum at different time points. The main processes were as followed: (1) complete drying of the slide chip, (2) preparation of cytokine standards, (3) blocking and incubation, (4) cleaning, (5) incubation and washing of the test antibody mixture, (6) Cy3-streptavidin incubation and cleaning, and (7) fluorescence detection. The RayBiotech QAR-CYT-2 data processing software was used to analyze the changes of the above inflammatory cytokine expressions in the development of the MDR PA-infected pneumonia.

### 2.6. Determination of the mRNA Expressions in the TLR4/MyD88/NF-*κ*B Signaling Pathway

The total RNA of rat lung tissue was extracted using the Tissue Total RNA Mini Kit following the protocol. The collected RNA was reverse transcribed according to the PrimeScript™ RT Master Mix reverse transcription system and its procedure. The mRNA expressions of TLR4, MyD88, NF-*κ*B, and ICAM-1 were detected by real-time PCR instrument (Cat. No: CFX96; Bio-Rad, USA). RT-qPCR conditions were initial denaturation for 30 seconds at 95°C, 40 cycles of amplification with denaturation at 95°C for 5 seconds, annealing at 60°C for 30 seconds. After 40 cycles, extension at 95°C for 10 seconds was done. The relative RNA expression was calculated by using the formula of 2^-*ΔΔ*Ct^ (*β*-actin for the internal reference). The primers were synthesized by Invitrogen Trading Co., Ltd. (Shanghai, China), and the primer sequences are exhibited in Table 1.

### 2.7. Statistical Analysis

SPSS 17.0 was used for statistical analysis and GraphPad prism 7.0 for drawing. The measurement data was presented as mean ± standard deviation. The statistical significance was evaluated using one-way ANOVA combined with the LSD multiple adjustment test.

## 3. Results

### 3.1. QGYD-LEV Improved the General State and Immune Organ Index of MDR PA-Infected Rats

**Figure 1:**
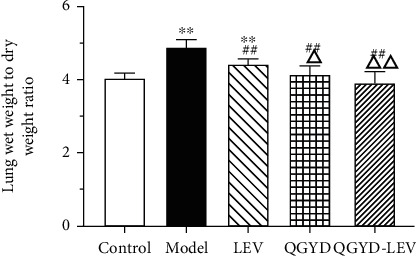
Effects of QGYD and LEV on lung wet weight to dry weight ratio (*W*/*D*) of MDR PA-infected rats. Bars represent mean ± SD (*N* = 8, per group). ^∗∗^*P* < 0.01, versus the Control group; ^##^*P* < 0.01, versus the Model group; ^△^*P* < 0.05, versus the LEV group; ^△△^*P* < 0.01, versus the LEV group.

**Figure 2:**
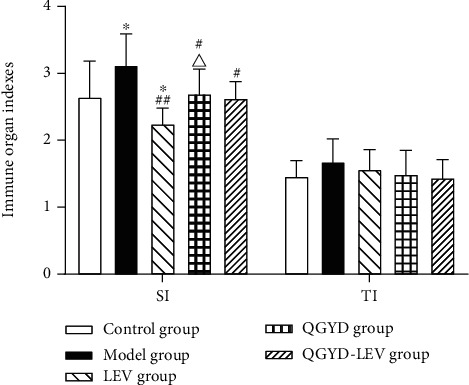
Effects of QGYD and LEV on spleen index (SI) and thymus index (TI) of MDR PA-infected rats. Bars represent mean ± SD (*N* = 8, per group). ^∗^*P* < 0.05, versus the Control group; ^#^*P* < 0.05, versus the Model group; ^##^*P* < 0.01, versus the Model group; ^△^*P* < 0.05, versus the LEV group.

In this study, we established a MDR PA-induced pneumonia model in SD rats by tracheal intubation. Our results showed that compared with the Control group, the rats in the model group had a poor state, which showed a slower response to the surrounding stimuli, various degrees of chills, more respiratory frequency and respiratory murmur, fewer diets, and body weight loss, as shown in Table 2. Moreover, the lung *W*/*D* (*P* < 0.01) and SI (*P* < 0.05) of the Model group were significantly higher, which indicated the occurrence of pulmonary edema and body immune response, as shown in Figures 1 and 2. In addition, the HE staining of lung tissue in the MDR PA-induced rat pneumonia model at different time points (1 d, 2 d, 3 d, and 5 d) was shown in Supplementary Figure 1. These results suggested that the development of pneumonia caused by MDR PA infection was accompanied with the activation of inflammatory immune response in rats.

LEV and QGYD singly or in combination were given for intervention during the experiment. The results showed that compared with the Model group, the drug intervention could improve the general state of MDR PA-infected rats and significantly inhibit the body weight loss and the increase of lung *W*/*D* (*P* < 0.01) and SI (*P* < 0.05) caused by MDR PA infection; in addition, the improving effect of QGYD-LEV was superior to that of LEV or QGY alone, as shown in Table 2 and Figures 1 and 2. TI also showed a similar trend, that is, the order from high to low was the Model group, LEV group, QGYD group, the Control group, and QGYD-LEV group; however, the differences among the five groups were not significant (*P* > 0.05), as shown in Figure 2. These results verified the efficacy of QGYD and LEV combination therapy on MDR PA-induced pneumonia.

### 3.2. Effects of QGYD-LEV on Inflammatory Cytokines in Rat Serum

**Figure 3:**
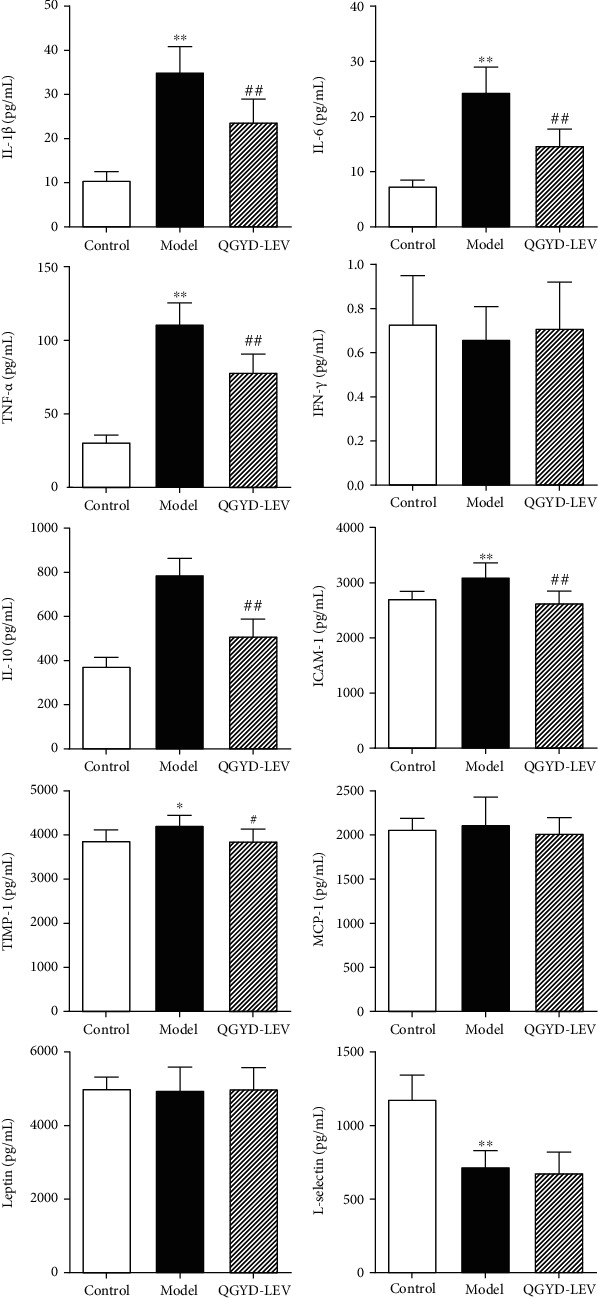
Effects of QGYD-LEV treatment for 5 days on serum inflammatory cytokines of MDR PA-infected rats. Bars represent mean ± SD (*N* = 8, per group). ^∗^*P* < 0.05, versus the Control group; ^∗∗^*P* < 0.01, versus the Control group; ^#^*P* < 0.05, versus the Model group; ^##^*P* < 0.01, versus the Model group.

Since inflammatory cytokines contribute to the occurrence and development of body inflammatory immune response [29, 30], we conducted the analyses by cytokine antibody array to find the inflammatory cytokines differentially expressed after QGYD-LEV treatment. As shown in Figure 3, the treatment of QGYD-LEV could significantly downregulate the increased expressions of IL-1*β*, IL-6, TNF-*α*, IL-10, ICAM-1, and TIMP-1 caused by MDR PA infection (*P* < 0.05).

**Figure 4:**
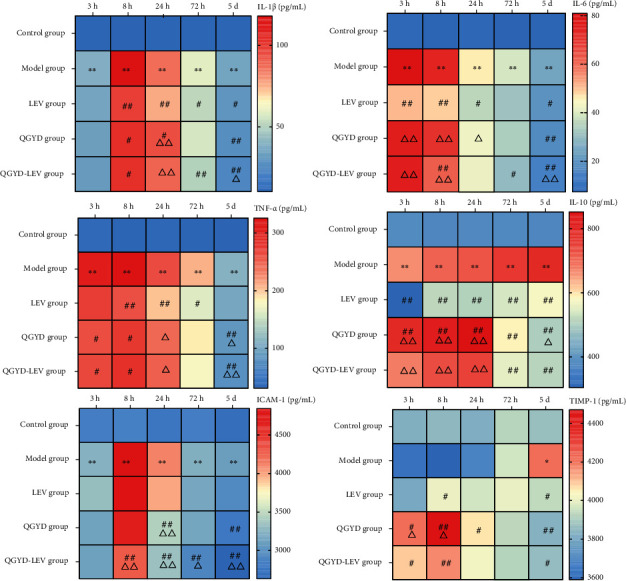
The differential cytokine profiles of six inflammatory cytokines in rat serum at different time points (*N* = 8, per group). ^∗^*P* < 0.05, versus the Control group; ^∗∗^*P* < 0.01, versus the Control group; ^#^*P* < 0.05, versus the Model group; ^##^*P* < 0.01, versus the Model group; ^△^*P* < 0.05, versus the LEV group; ^△△^*P* < 0.01, versus the LEV group.

Based on this, we then further studied the changes of the above six cytokines at different time points after modeling. As shown in Figure 4, we observed higher inflammatory cytokine expressions in the Model group than the Control group at most time points (*P* < 0.05). These results demonstrated that MDR PA infection could lead to significant dynamic changes in IL-1*β*, IL-6, TNF-*α*, IL-10, and ICAM-1 expressions and break their original balance.

Compared with the Model group, the expressions of IL-1*β*, IL-6, TNF-*α*, and IL-10 significantly decreased from 3–8 h after the LEV intervention (*P* < 0.01). Moreover, the cytokine expressions of IL-1*β*, IL-6, TNF-*α*, and IL-10 were higher at 8–24 h and lower at 5 d after the QGYD intervention (QGYD and QGYD-LEV) in comparison to the LEV group (*P* < 0.05), while the ICAM-1 expression was consistently lower (*P* < 0.05). In addition, we observed a higher anti-inflammatory cytokine IL-10 level at 3-24 h and lower level at 72 h-5 d in the QGYD group and QGYD-LEV group than the Model group (*P* < 0.01); however, the differences at 3-24 h between the Model group and QGYD-LEV group were not significant. These results suggested the efficacy of QGYD was related to its regulation of the inflammatory cytokine imbalance caused by MDR PA infection.

### 3.3. Inhibitory Effect of QGYD-LEV on the TLR4/MyD88/NF-*κ*B Signaling Pathway in MDR PA-Infected Rats

**Figure 5:**
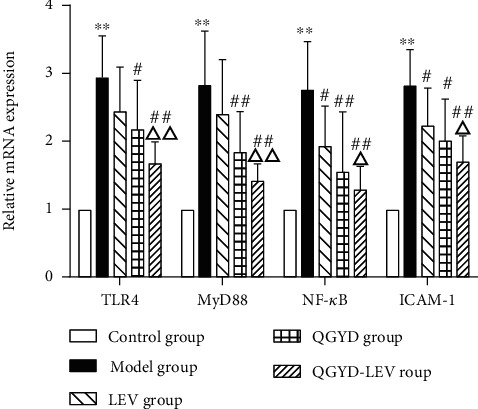
The mRNA expressions of TLR4, MyD88, NF-*κ*B, and ICAM-1 in rat lung tissue were detected by RT-qPCR. Bars represent mean ± SD (*N* = 8, per group). ^∗∗^*P* < 0.01, versus the Control group; ^#^*P* < 0.05, versus the Model group; ^##^*P* < 0.01, versus the Model group; ^△^*P* < 0.05, versus the LEV group; ^△△^*P* < 0.01, versus the LEV group.

To further evaluate the effect of QGYD on inflammatory immune response, the mRNA expressions of the key genes of the TLR4/MyD88/NF-*κ*B signaling pathway and ICAM-1 of the downstream in lung tissue were assayed by RT-qPCR. As shown in Figure 5, our results showed that compared with the Control group, the expressions of TLR4, MyD88, NF-*κ*B, and ICAM-1 of the Model group were significantly higher (*P* < 0.01), indicating that the TLR4/MyD88/NF-*κ*B signaling pathway was activated during the development of MDR PA-induced pneumonia in rats. QGYD intervention (especially QGYD-LEV) could significantly downregulate the increase of TLR4, MyD88, NF-*κ*B, and ICAM-1 mRNA expressions caused by MDR PA infection (*P* < 0.05), revealing that QGYD-LEV treatment could inhibit the TLR4/MyD88/NF-*κ*B signaling pathway in MDR PA-infected rats.

## 4. Discussion

A monitoring report from China Antimicrobial Resistance Surveillance System showed that the susceptibility of PA to almost all antibiotics had declined and the resistance rate to commonly used antibacterial drugs remained at a high level [31], which has made the clinical treatment of PA infection facing severe challenges. LEV was once an effective antibiotic against PA. However, with its widespread use, PA has developed new drug resistance mechanisms and even multidrug resistance mechanisms in a single strain, which renders even the most effective drugs ineffective [32, 33].

Traditional Chinese medicine has unique advantages in treating infectious diseases. Syndrome differentiation treatment guided by the theory of traditional Chinese medicine can combine bacteriostasis and sterilization with the body protection and the immune regulation, thereby reducing the damage caused by drug-resistant PA infection. In clinical treatment of anti-infection, the effects of many traditional Chinese medicines and their compounds combined with antibiotics have been gradually recognized [34, 35]. QGYD is a clinical efficacious prescription of Professor Qingquan Liu from Beijing Chinese Medicine Hospital, the formation of which is based on the understanding of traditional Chinese medicine core pathogenesis of drug-resistant PA infection combined with years of practical experience. In this study, we verified the efficacy of QGYD-LEV therapy on a rat model of MDR PA-induced pneumonia and discussed its mechanism from a view of the regulation of inflammatory immune response.

The results of this study showed that based on LEV in the treatment of MDR PA-infected rats, the combination with QGYD could improve the general states and increase the body weight. The results of lung *W*/*D* and immune organ indexes indicated that QGYD-LEV therapy could effectively regulate and control the deterioration of MDR PA infection. Lung *W*/*D* is one of the most common indicators to characterize lung tissue injury by reflecting lung water content and the severity of pulmonary edema, and its rise represents increased pulmonary capillary permeability and worse pulmonary edema [36]. Lung *W*/*D* of the infected rats significantly decreased after QGYD-LEV treatment, which was superior to LEV alone in alleviating pulmonary edema. The functions of spleen and thymus are associated with body immunity [37, 38]. SI and TI, as preliminary observation indexes to characterize body immune function, can be used to evaluate the effects of drugs on immune organs and also can indirectly reflect the quantity of lymphocytes in immune organs [39]. QGYD-LEV therapy improved the increase of SI and TI caused by MDR PA infection and the decline caused by LEV, which suggested that QGYD could be an immunomodulator to the body's inflammatory immune response.

Based on the above-mentioned results, cytokine antibody array and RT-qPCR were applied to further explore the mechanism of QGYD restoring LEV susceptibility to MDR PA by investigating the impact of QGYD on inflammatory cytokines and the TLR4/MyD88/NF-*κ*B signaling pathway.

Inflammatory cytokines play a crucial role in the body's immune regulatory network, mediating inflammation, regulating immune response, and participating in tissue repair [40–42]. In this study, we found six inflammatory cytokines differentially expressed in MDR PA-infected rats after QGYD-LEV treatment, including proinflammatory cytokines IL-1*β*, IL-6, TNF-*α*, anti-inflammatory cytokine IL-10, adhesion molecule ICAM-1, and chemokine TIMP-1. In addition, MDR PA infection led to significant dynamic changes of the above cytokines except TIMP-1 at 3-72 h.

Proinflammatory and anti-inflammatory cytokines maintain a dynamic balance under normal conditions while infections can cause the imbalance, which may be key to the pathogenesis of inflammatory diseases. The results of the cytokine antibody array showed that the proinflammatory cytokines IL-1*β*, IL-6, and TNF-*α* in rat serum were significantly increased in the early stage of MDR PA infection, and the intervention of LEV could reduce the release of these three proinflammatory cytokines. Compared with LEV alone in the treatment, the intervention of QGYD alone or combined could increase the release of these proinflammatory cytokines to some extent in the early stage of infection and rapidly decrease their release in the later stage. These proinflammatory cytokines can induce the production of adhesion molecules, such as ICAM-1, which are involved in the occurrence of tissue injury and the regulation of inflammatory immune response [43, 44]. In this study, the adhesion molecule ICAM-1 was significantly downregulated after QGYD-LEV treatment, while LEV treatment had no significant effect on ICAM-1. Inflammation is a defensive response that the body produces when it fights against pathogenic bacteria. Moderate inflammation is beneficial and protective to the body, while excessive and insufficient inflammation is both harmful [45]. Precisely, excessive inflammation can cause tissue damage and organ dysfunction, while insufficient inflammation is not conducive to the body's elimination of infectious agents, toxins released by bacteria, and damaged tissues. Compared with the excessive inflammation in the Model group and the insufficient inflammation in the LEV group, QGYD and QGYD-LEV treatment maintained the inflammation at a moderate level in the early stage of MDR PA infection, which could ensure the body function normally on the elimination of pathogenic bacteria and toxins, control the inflammation, and avoid cascade waterfall effects and serious self-injury. Moreover, the release of proinflammatory cytokines rapidly reduced in the later stage after QGYD and QGYD-LEV treatment, which could prevent the body regulatory from being imbalanced that caused by the prolonged inflammation.

In the process of pneumonia induced by MDR PA infection, the anti-inflammatory cytokine IL-10 in the Model group increased from the early stage and continued to increase until it reached a peak in the later stage, which was consistent with the report that the expression of IL-10 was later than proinflammatory cytokines in the mice challenged by LPS [46]. The expression of IL-10 was significantly inhibited in the LEV group, compared with that in the Model group. QGYD and QGYD-LEV treatment could promote the release of IL-10 and make the peak appear earlier. Furthermore, the release of IL-10 in the QGYD group and QGYD-LEV group decreased rapidly in the later stage of infection. The growth and decline of both proinflammatory and anti-inflammatory cytokines determine the development, transformation, and prognosis of inflammation [47]. In order to avoid harmful effects of the inflammation after it produces a beneficial effect in the early stage, the body compensatively releases anti-inflammatory cytokines to protect normal tissues from inflammatory damage. It has been confirmed that excessive or insufficient release of IL-10 is associated with poor prognosis [48, 49]. Overexpression of IL-10 can inhibit the release of the proinflammatory cytokines, obstruct the elimination of bacteria and toxins, and thus lead to an immunosuppressive effect, while the underexpression can cause persistent and excessive proinflammatory response and thus induce systemic inflammatory response syndrome [50]. In this study, compared with the overexpression of IL-10 in the Model group and the underexpression in the LEV group, QGYD-LEV treatment significantly increased the release of IL-10 in the early stage of infection to control the proinflammatory response at a moderate level. Moreover, it rapidly reduced the expression of IL-10 in the later stage with the decline of IL-1*β*, IL-6, TNF-*α*, and IFN-*γ*, to prevent the continuously excessive release of IL-10 from causing severe immune suppression and uncontrollable infection. These could benefit the body from controlling the inflammation and maintaining the stability of internal environment during MDR PA infection.

The TLR4/MyD88/NF-*κ*B signaling pathway can regulate the expressions of various inflammatory cytokines, initiate natural and acquired immunity against pathogens, and participate in pathological processes such as inflammatory cell chemotaxis and cell adhesion [51]. It was reported that the TLR4/MyD88/NF-*κ*B signaling pathway was involved in the inflammatory immune response of multiple diseases [52–55]. Therefore, it has become increasingly important to regulate the TLR4/MyD88/NF-*κ*B signaling pathway in the prevention and treatment of inflammatory and immune-related diseases. The results of RT-qPCR revealed that MDR PA infection could activate this signaling pathway in rats and significantly upregulate the mRNA expressions of TLR4, MyD88, NF-*κ*B, and ICAM-1. Moreover, compared with LEV alone, QGYD-LEV therapy could significantly reduce and even normalize the mRNA expressions of the key genes of the TLR4/MyD88/NF-*κ*B signaling pathway. However, further studies are needed to explore the effect of QGYD and LEV on MDR PA-infected rats at the protein level. In addition, some transcription factors and their inhibitors of this signaling pathway can also play an important role in the body's inflammatory immune response [56–61]. Therefore, the anti-MDR PA mechanism of QGYD-LEV therapy needs to be verified in more interference experiments.

## 5. Conclusions

In conclusion, QGYD-LEV treatment could promote the recovery of MDR PA-infected rats from a disorder of inflammatory immune response to equilibrium by regulating inflammatory cytokines and the mRNA expressions of the TLR4/MyD88/NF-*κ*B signaling pathway, which provides evidence for its clinical application to MDR PA infection.

## Figures and Tables

**Table 1:** Sequences of primers used in the RT-qPCR analysis.

Genes	Orientation	Primer sequence (5′-3′)
TLR4	Forward	5′-GGCATCATCTTCATTGTCCTTG-3′
	Reverse	5′-AGCATTGTCCTCCCACTCG-3′
MyD88	Forward	5′-CAACCAGCAGAAACAGGAGTCT-3′
	Reverse	5′-ATTGGGGCAGTAGCAGATGAAG-3′
NF-*κ*B	Forward	5′-GCAAACCTGGGAATACTTCATGTGACTAAG-3′
	Reverse	5′-ATAGGCAAGGTCAGAATGCACCAGAAGTCC-3′
ICAM-1	Forward	5′-ACCAGACCCTGGAGATGGAGA-3′
	Reverse	5′-ACCGTGGGCTTCACACTTCA-3′
*β*-Actin	Forward	5′-GCCATGTACGTAGCCATCCA-3′
	Reverse	5′-GAACCGCTCATTGCCGATAG-3′

**Table 2:** Effects of QGYD and LEV on rat body weight in 5 days (*N* = 8, per group).

Group	Body weight (g)				
	1 d	2 d	3 d	4 d	5 d
Control	242.3 ± 5.6	235.4 ± 6.9	242.0 ± 7.2	248.4 ± 6.9	254.4 ± 8.1
Model	243.1 ± 11.4	219.9 ± 10.0^∗∗^	211.0 ± 10.0^∗∗^	215.8 ± 9.5^∗∗^	218.9 ± 9.1^∗∗^
LEV	243.9 ± 6.9	223.4 ± 5.1^∗∗^	220.7 ± 4.4^∗∗#^	229.5 ± 4.9^∗∗##^	234.4 ± 3.5^∗∗##^
QGYD	242.7 ± 8.6	222.3 ± 7.0^∗∗^	226.1 ± 7.5^∗∗##^	234.7 ± 9.0^∗∗##^	241.5 ± 8.3^∗∗##^
QGYD-LEV	243.0 ± 7.4	225.1 ± 7.1^∗∗^	230.6 ± 7.9^∗∗##△^	240.5 ± 7.1^∗##△△^	248.7 ± 8.1^##△△^

Note: ^∗^*P* < 0.05, versus the Control group; ^∗∗^*P* < 0.01, versus the Control group; ^#^*P* < 0.05, versus the Model group; ^##^*P* < 0.01, versus the Model group; ^△^*P* < 0.05, versus the LEV group; ^△△^*P* < 0.01, versus the LEV group.

## Data Availability

The data used to support the findings of this study are available from the corresponding authors upon request.

## Supplementary Materials

Supplementary Materials Supplementary Figure 1 HPLC fingerprint of three different samples of QGYD extract.
