# Supplementary material for: Qiguiyin Decoction Improves Multidrug-Resistant Pseudomonas aeruginosa Infection in Rats by Regulating Inflammatory Cytokines and the TLR4/MyD88/NF-κB Signaling Pathway
Source: Biomed Res Int. 2022 Jan 13;2022:5066434. doi: 10.1155/2022/5066434 (PMC8776462; doi:10.1155/2022/5066434)
Supplement: Supplementary Materials — Supplementary Figure 1 HPLC fingerprint of three different samples of QGYD extract. [file 5066434.f1.pdf]

Supplementary Figure

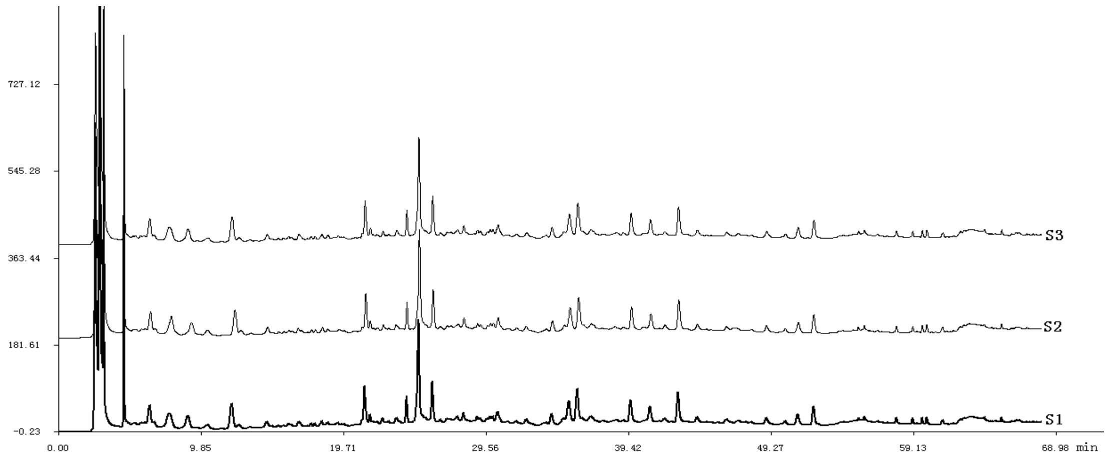

Supplementary Figure 1 HPLC fingerprint of three different samples of QGYD extract. Ultrasonic extraction was used to sample preparation for HPLC fingerprint. Briefly, QGYD extract (1.000 g) was accurately weighed and it was extracted with 70% methanol (25 mL) for 20 min, then weighed, next added 70% methanol to make up for the lost weight. The sample solution was filtered through a 0.45 μm membrane before HPLC analysis. The analysis was performed on a Waters Liquid Chromatography, equipped with Waters 1525 solvent infusion pump, Waters 2489 UV detector and Waters breeze 2 chromatography workstation. The samples were separated on a Kromasil-C18 column (4.6 mm×250 mm, 5 μm). The mobile phase was composed of acetonitrile solution (A) and 0.05% phosphoric acid aqueous solution (B). The gradient elution program was as follows: 98%-97% (B) for 0–5 min; 97%–83% (B) for 5–22 min; 83%–82% (B) for 22–27 min; 82%–68% (B) for 27–47 min; 68%–37% (B) for 47–57 min; 37%–0% (B) for 57–63 min. The injection volume was 10 μL, the flow rate was 1.0 mL/min and the column temperature was 25°C. The detector was set to 203 nm.
